# Stressors Due to Handling Impair Gut Immunity in Meagre (*Argyrosomus regius*): The Compensatory Role of Dietary L-Tryptophan

**DOI:** 10.3389/fphys.2019.00547

**Published:** 2019-05-10

**Authors:** Gloria Asencio-Alcudia, Karl B. Andree, Inmaculada Giraldez, Dariel Tovar-Ramirez, Alfonso Alvarez-González, Marcelino Herrera, Enric Gisbert

**Affiliations:** ^1^ Centro de Investigaciones Biológicas del Noroeste (CIBNOR), La Paz, Mexico; ^2^ Laboratorio de Acuicultura Tropical, División Académica de Ciencias Biológicas, Universidad Juárez Autónoma de Tabasco (DACBiol-UJAT), Villahermosa, Mexico; ^3^ Programa de Cultius Aquàtics, IRTA, Centro de San Carlos de la Ràpita (IRTA-SCR), Tarragona, Spain; ^4^ Faculty of Experimental Sciences, University of Huelva, Huelva, Spain; ^5^ IFAPA Centro Agua del Pino, Cartaya, Spain

**Keywords:** *Argyrosomus regius*, amino acid, stress, immune response, gene expression, aquaculture, diet supplement

## Abstract

In the context of intensive aquaculture, meagre (*Argyrosomus regius*) is one of the most important new aquaculture species in Southern Europe and several studies are focused on the optimization of its culture. Nevertheless, stressors such as handling during transport or culture maintenance may affect the immune system, thereby impairing some immune responses or provoking cellular damage. One strategy that has been used to avert this type of negative stress response is the supplementation of amino acids to improve resistance to stress. In this experiment, meagre (105.0 ± 2.6 g, mean ± standard deviation) juveniles were fed two diets for a period of 7 days, the first a commercial diet supplemented with 1% tryptophan (Trp) and second, the same commercial diet without tryptophan supplementation (control group). The effects of two types of handling stressors (air exposure and confinement/netting) on fish fed both diets was evaluated in terms of gene expression of the selected gut immunity markers, such as (1) innate immune response processes: *c3 complement* (*c3*), *lysozyme* (*lys*), and *cyclooxygenase* (*cox2*); (2) humoral immune response processes: *interferon type 1* (*ifn1*), *mx protein* (*mxp*), *interleukin 1b* (*il-1b*), *tumor necrosis factor 1a* (*tnf1a*), and *interleukin 10* (*il-10*); (3) antimicrobial peptides: *defensin* (*def*), *hepcidin* (*hep*), *piscidin* (*pis*), and a marker for mitochondrial respiration: *glyceraldehyde 3-phosphate dehydrogenase* (*gapdh*). Samples of the anterior intestine were collected at 1 and 6 h post-stress (hps). Results showed that in fish fed 1% Trp, the air exposure resulted in an upregulation of gene expression at 6 hps for *c3*, *lys*, *cox2*, *ifn1*, *mxp*, *il-10* and *gapdh*, and *il-1b* and *pis*. The confinement/netting test for fish fed 1% Trp resulted in an upregulation of *c3* and *mxp* and a downregulation of *cox2*, *ifn1*, *il-1b*, *tnf1a*, *il-10*, *def*, *hep*, and *gapdh* at both post-stress times (1 and 6 hps). According to the present study, dietary supplementation with 1% Trp may be considered as a proper nutritional strategy for improving tolerance and/or alleviating acute response to handling stressors.

## Introduction

Interest in animal welfare has increased in the last decade because of ethical or legal concerns, as well as its effect on growth performance and product quality (Ashley, 2007; Noble et al., 2012; Toni et al., 2019). In this context, routine aquaculture procedures often involve fish manipulation outside water for a period of time, thereby causing stress, immunosuppression, general discomfort, or even mortality in the case of extended manipulation and exposure times. Under stressful conditions, the immune system can become depressed accentuating the risk for infectious diseases (Barton, 2002; Kumar et al., 2012; Gisbert et al., 2018; Khansari et al., 2018). Consequently, recent advances in the field of aquafeed formulation have led to the development of new feed strategies, using so-called “functional feeds,” to enhance the sustainability of fish production, concerning the growth, survival, and health of the animal by reducing the stress imposed during different handling processes and/or modulating immune functions (Olmos-Soto et al., 2015). In this sense, different nutritional studies have demonstrated that dietary supplementation with essential amino acids, which are precursors to other significant biomolecules, i.e., biogenic amines, heme, or purine and pyrimidine groups (Nelson and Cox, 2013; Andersen et al., 2016), can improve several physiological functions that regulate key metabolic pathways in the organism thus improving survival, development, growth, health, welfare, and reproduction in different vertebrates, including fish species (Li et al., 2009).

Among them, several studies have shown that tryptophan (Trp) enhanced survival and growth by decreasing aggressive behavior, mitigating crowding stress, and improving post-stress recovery in different fish species (Hseu et al., 2003; Höglund et al., 2005; Hoseini et al., 2012; Herrera et al., 2017; Azeredo et al., 2017a). As Trp is a substrate for serotonin and melatonin, it can be expected to have a significant role in establishing behavioral states; furthermore, it is also a substrate for nicotinamide adenine dinucleotide (NAD) synthesis; so, there are major implications for electron transport and energy generation in the cell (Richard et al., 2009). In addition, this essential α-amino acid has also been shown to have significant impacts on immune functions controlled by specific pathways, *via* its role as a structural component of specific transcription factors (Mamane et al., 1999). More recently, meagre (*Argyrosomus regius*) fed with a diet supplemented with Trp modulated its serological immune parameters after stress caused by air exposure (González-Silvera et al., 2018).

With regard to the abovementioned considerations of diet supplementation, the impact of animal handling on stress and systemic immunity has been widely studied in livestock production and aquaculture practices (Barton, 2002; Kumar et al., 2012), whereas limited information is available about the impact of routine animal handling on the immunity of mucosal tissues, and more specifically the intestine (Schokker et al., 2015). The mucosal immunology of the gut is key in not only maintaining a balanced response toward the commensal bacteria that are a normal part of the microbiota but also the prevention of growth of pathogens. Many immune genes are involved in this complex process (Gómez et al., 2013; Ostaff et al., 2013). Moreover, the physiological response to stress is similar between vertebrates and teleost fish where two pathways of response to stress stimulation are activated: the brain-sympathetic-chromaffin cell axis, responsible for a quick release of circulating catecholamine and the hypothalamic-pituitary-interrenal axis (HPI) in charge of the release of an endocrine cascade, which leads to secondary responses that induce changes in cellular metabolites that ultimately alter immune gene expression in fish (Wendelaar-Bonga, 1997; Barton, 2002). However, the gut-associated lymphoid tissue (GALT) in fish is quite different from that of mammals (Foey and Picchietti, 2014). While some general features of the mucosal immunity of the gut is shared with other better studied vertebrates (Gómez et al., 2013), there are unique features such as a class of immunoglobulins, Ig T that replaces the Ig E normally associated to mucosal immunity in higher vertebrates (Zhang et al., 2010; Mutoloki et al., 2015). In addition, chronic stress increases cortisol levels to a point at which the stress impacts negatively on the overall immune responses (Richard et al., 2009). It is well known that the immune system of the gut is responsive to oral vaccination (Mutoloki et al., 2015), but the responsiveness of gut immunity to different stressors is of special relevance. Vaccination protocols require significant handling, and negative consequences from the handling stressors may also influence the effectiveness of the administered vaccine. It is toward this end that we evaluated inclusion of Trp in the diet of fish groups receiving different types of stress and evaluate the concomitant immune response at different levels: (1) innate immune response and (2) endocrine signaling of effector cells. It is in this context that it is interesting to reduce the negative impacts of stress through dietary supplements.

Therefore, the objective of the present study was to evaluate the impact of two common aquaculture handling stressors like air exposition and confinement/netting on local gut immunity in fish fed a diet supplemented with 1% Trp compared to another group of fish fed the same diet, but not supplemented with this essential amino acid. To achieve the abovementioned goal, the following gut immunity markers were used to evaluate the potential stressor-alleviating effects of Trp on local gut immunity: (1) innate immune response processes: *c3 complement* (*c3*), *lysozyme* (*lys*), and *cyclooxygenase* (*cox2*); (2) humoral immune response processes: *interferon type 1* (*ifn1*), *mx protein* (*mxp*), *interleukin 1b* (*il-1b*), *tumor necrosis factor 1a* (*tnf1a*), and *interleukin 10* (*il-10*); (3) antimicrobial peptides: *defensin* (*def*), *hepcidin* (*hep*), *piscidin* (*pis*), and a marker for mitochondrial respiration: *glyceraldehyde 3-phosphate dehydrogenase* (*gapdh*).

## Materials and Methods

### Fish Maintenance and Experimental Diets

Fish used in this trial (*n* = 150) were obtained from the Olhão Fish Farming Pilot Station (EPPO) of the Portuguese Institute of Atmosphere and the Sea (EPPO-IPMA, Olhão, Portugal) and kept in four re-circulating seawater tanks (600 L; 25 fish each) in the IFAPA Centro Agua del Pino (Cartaya, Spain). Fish body weight (BW) was 105.0 ± 2.6 g (mean ± standard deviation). Water temperature was maintained at 19.0 ± 1.0°C with a flow rate of 2 m^3^ h^−1^ and 33 PSU. The photoperiod was natural (N37°13′4.31″), and fish were fed a commercial pelleted diet (L4-Alterna, Skretting, Burgos, Spain; 47% protein, 20% lipid, 7% ash, 4% cellulose, 1% total phosphorus, and 18 MJ kg^−1^ energy) at a feeding rate of 1% BW day^−1^. Fish were allowed to acclimatize to experimental conditions for 21 days before the start of the trial.

For the study, the same commercial diet that was used during the acclimation process was also used for the nutritional phase of the study. In particular, pellets were finely ground and then mixed with water (400 ml kg^−1^ dry feed) and Trp (Ref. T0254; Sigma-Aldrich, St. Louis, USA) was added in order to achieve a final concentration of 1% dry weight. Then, the mixture was pelleted into 2 mm diameter and 20–25 cm length strips that were dried at 60°C for 24 h, and manually cut into 2–3 mm size pellets. At the same time, half of the commercial feed was re-pelleted without Trp supplementation, and this batch of feed was used as a control diet. Both control (C) and experimental (T) diets were stored at 4°C until use.

### Experimental Design

Fish were fed both diets for 7 days (two tanks per treatment) prior to evaluating the impact of handling stress on local gut immunity. Two different types of stressors were used as previously described by González-Silvera et al. (2018). An air exposition stress treatment (A), in which all fish from each experimental tank were netted and kept out of the water for 3 min, and then returned to their respective experimental tanks; and a confinement/netting stress treatment (N), in which water levels in experimental tanks were reduced (until 20 cm) and then, fish were chased with a net (without air exposure) for 3 min. This procedure was repeated every 10 min for 1 h. In both treatments (A and N), gut samples were collected at 1 and 6 h post-stress (1H and 6H). In order to evaluate whether stressors had an impact on local gut immune function in meagre, and to set up a baseline for comparative studies, samples from each experimental group (C and T = control and tryptophan diets, respectively) were taken prior to stress episodes (B = baseline).

For tissue sampling purposes, fish from both treatments were sacrificed at different sampling points (B, 1H, and 6H) with an overdose of anesthetic (>1 ml L^−1^ 2-phenoxyethanol, Sigma, USA), their digestive system dissected and a portion of the anterior intestine (*ca.* 1 cm in length) was obtained from each fish (*n* = 6 per sampling point and experimental condition). This portion of the gut included the intestinal mucosa, submucosa, and *muscularis*, since the thin layer of smooth musculature could not be removed from gut samples. Then, tissue samples were fixed at 4°C for 24 h in RNAlater® (Invitrogen, Waltham, USA) and frozen at −80°C until analysis (RNA extraction).

The experiment complied with the Guidelines of the European Union Council (2010/63/EU) and the Spanish Government (RD1201/2005; RD53/2013 and law 32/2007) for the use of laboratory animals. All experimental protocols were approved by the Ethical Committee of the IFAPA (Seville, Spain).

### Total RNA Extraction and cDNA Synthesis

Total RNA from the anterior intestine was extracted using the RNeasy Mini kit (Qiagen, Hilden, Germany) according to the manufacturer’s instructions. RNA concentration and purity were determined by spectrophotometry (NanoDrop2000, Thermo Fisher Scientific, Madrid, Spain) measuring the absorbance at λ = 260 and 280 nm. The integrity of extracted RNA was verified with visualization of the 28S and 18S ribosomal RNA bands by electrophoretic separation on a 1.2% agarose gel. For cDNA synthesis, RNA was reverse transcribed in a 20 μl reaction volume containing 2 μg total RNA using SuperScript™ First-Strand Synthesis System for RT-PCR (Invitrogen, Waltham, USA) with oligo (dT) (0.5 μg μl^−1^), random hexamer primers (50 ng μl^−1^) and 10 mmol L^−1^ dNTP mix, 10× RT buffer [200 mmol L^−1^ Tris-HCl (pH = 8.4), 500 mmol L^−1^ KCl], 25 mmol L^−1^ MgCl_2_, 0.1 mol L^−1^ DTT, RNaseOUT (40 U μl^−1^), and SuperScript™ II RT, followed by RNAse H treatment. The resulting cDNA was diluted 1:20 in DEPC-treated water, and the prepared dilutions were used for gene expression analysis.

### Real-Time qPCR

The qPCR reactions were performed for the six individuals per treatment in duplicate on a CFX96 Touch™ Real-Time PCR Detection System (Bio-Rad, Madrid, Spain). Each reaction (20 μl) contained 10 μl SsoAdvanced™ Universal SYBR® Green Supermix (Life Technologies, Carlsbad, California, USA), 0.50 μl forward primer, 0.50 μl reverse primer, 2 μl of cDNA sample, and 7 μl of RNase/DNase-free water. The real-time qPCR protocol started with 95°C for 3 min, followed by 40 cycles of 15 s at 95°C followed by 30 s at the specific annealing temperature for each primer pair followed by 30 s at 72°C with a final melt curve step to establish standard melt curve profiles for each amplicon. Relative expression was calculated using CFX Manager 3.0 (Bio-Rad, Madrid, Spain) software, with elongation factor 1 alpha (EF1α) and hypoxanthine-guanine phosphoribosyltransferase (HPRT) as endogenous controls, while basal (pre-stress) samples from control and Trp groups were used for normalizing the relative quantification within each experimental diet group. All samples were run in duplicate with a negative control (no RT enzyme) included to confirm absence of genomic DNA contamination and an additional negative control on each plate containing no template cDNA.

### Statistical Analysis

To determine differences between gene expression of experimental diets and the time of exposure to stress, all data were checked for normality (Kolmogorov test) and homoscedasticity (Levene test), and analyzed by two-way ANOVA using Tukey as a *post hoc* test, with a significance level of *p* < 0.05 using the statistical software Statistica 7 (StatSoft, Inc., USA). These results were plotted using a surface response analysis for clearer visualization. A heatmap for visualizing different patterns in gene expression was also conducted by means of GraphPad Prism version 7 (GraphPad Software, La Jolla, California, USA). In order to evaluate whether both tested stressors had an impact on local gut immunity in fish fed the C diet, gene expression levels in gut samples from fish prior to stress (CB) and samples taken 1 h after both stress treatments (CA1H and CN1H) were compared by means of the relative expression software tool (REST) analysis 2009 (v2.0.13). Finally, a principal component analysis (PCA) was used to discriminate gene expression, to identify the treatments associated with 1% Trp supplementation and stressors and to minimize the influence of inter-individual variations.

## Results

**Figure 1:**
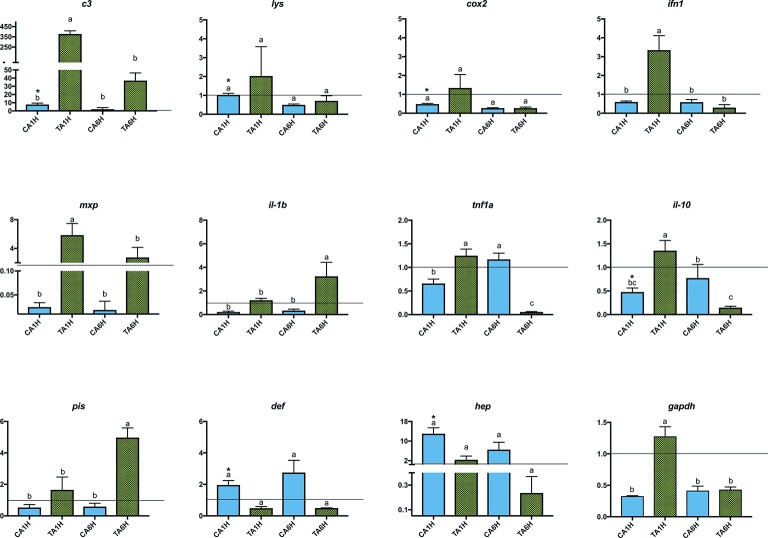
Gene expression levels in gut-associated lymphoid tissue (GALT) after air exposure stressor at 1 and 6 h post-stress. Quantitative PCR of specific mRNA markers of innate immune response processes (*c3 complement protein*, *lysozyme*, *cox2*, *mxp*, *piscidin*, *defensin,* and *hepcidin*), immune cytokines (*ifn*, *il-1b*, *tnf1a*, and *il-10*) and *gapdh,* normalized with elongation factor 1a and hypoxanthine-guanine phosphoribosyltransferase (*n* = 6 per experimental group). Asterisk shows differences between control without stress (resting fish) and control samples after 1 h of air exposure stress test without 1% Trp supplementation (REST analysis), whereas different letters denote statistically significant differences between experimental groups (two-way ANOVA). C, control feed; T, 1% Trp feed; A, air exposure stressor; 1H/6H, 1/6 h post-stress.

**Figure 2:**
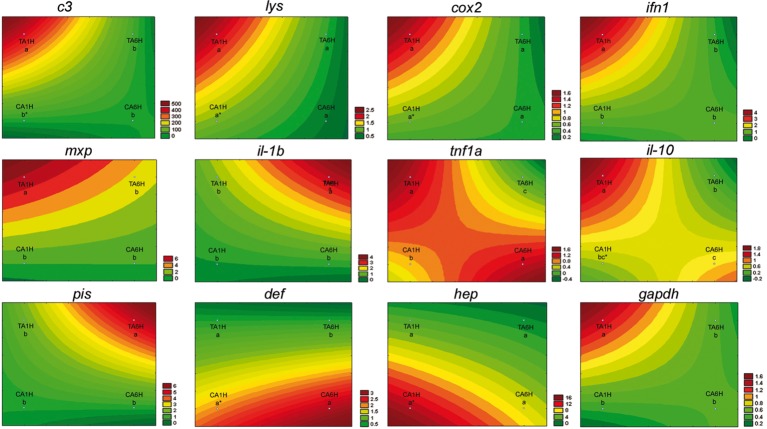
Visualization of gene expression results in gut-associated lymphoid tissue (GALT) after air exposure stressor at 1 and 6 h post-stress by means of response surface analysis. Quantitative PCR of specific mRNA markers of innate immune response processes (*c3 complement protein*, *lysozyme*, *cox2*, *mxp*, *piscidin*, *defensin*, and *hepcidin*), immune cytokines (*ifn*, *il-1b*, *tnf1a*, and *il-10*) and *gapdh,* normalized with elongation factor 1a and hypoxanthine-guanine phosphoribosyltransferase (*n* = 6 per experimental group). Asterisk shows differences between control without stress (resting fish) and control samples after 1 h of air exposure stress test without 1% Trp supplementation (REST analysis), whereas different letters denote statistically significant differences between experimental groups (two-way ANOVA). C, control feed; T, 1% Trp feed; A, air exposure stressor; 1H/6H, 1/6 h post-stress.

In the present study, gene expression of several key genes of the innate and humoral immune system was assessed in *A. regius* juveniles fed C and T diets before and after air exposure (A) and confinement/netting stressors (N). Regarding the air exposure stress, an upregulation in gene expression (asterisks) was found between gut samples taken before the stress episode and at 1 hps for *c3*, *lys*, *cox2*, *il-10*, *def*, and *hep* genes in fish fed the C diet (REST analysis, *p* < 0.05). Regarding the results from fish fed the T diet, several genes (*lys*, *cox2*, *def*, and *hep*) did not show statistical differences (*p* > 0.05) in gene expression patterns when fish were stressed by air exposure. However, *gapdh*, *c3 complement*, *ifn1*, *mxp*, *tnf1a*, and *il-10* showed the highest expression due to air exposure (*p* < 0.05) at 1 hps (TA1H) and the lowest expression at 6 hps (TA6H) in fish fed with the 1% Trp supplementation, while *il-1b* and *pis* showed the highest expression due to air exposure (*p* < 0.05) at 6 hps (TA6H) when fish were fed the Trp diet (Figures 1, 2).

**Figure 3:**
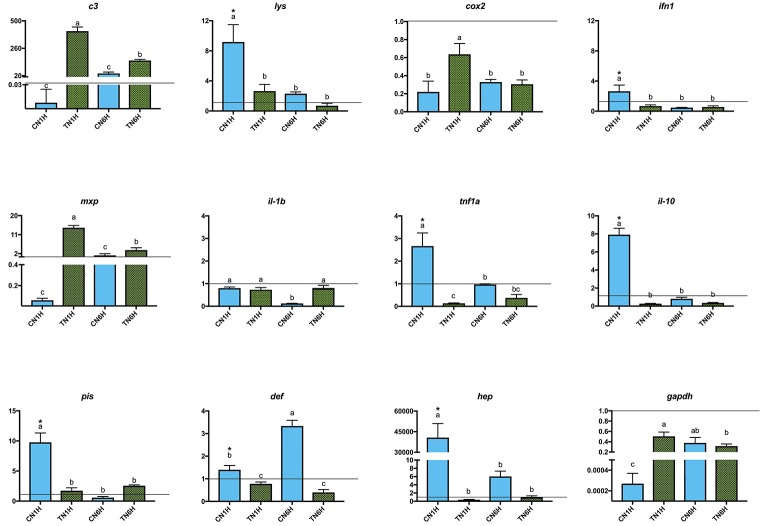
Gene expression levels in gut-associated lymphoid tissue (GALT) after netting-handling stress at 1 and 6 h post-stress. Quantitative PCR of specific mRNA markers of innate immune response processes (*c3 complement protein*, *lysozyme*, *cox2*, *mxp*, *piscidin*, *defensin*, and *hepcidin*), immune cytokines (*ifn*, *il-1b*, *tnf1a*, and *il-10*) and *gapdh*, normalized with elongation factor 1a and hypoxanthine-guanine phosphoribosyltransferase (*n* = 6 per experimental group). Asterisk shows differences between control without stress (resting fish) and control samples after 1 h of air confinement/netting stress test without 1% Trp supplementation (REST analysis), whereas different letters denote statistically significant differences between experimental groups (two-way ANOVA). C, control feed; T, 1% Trp feed; N, netting stress; 1H/6H, 1h/6 h post-stress.

**Figure 4:**
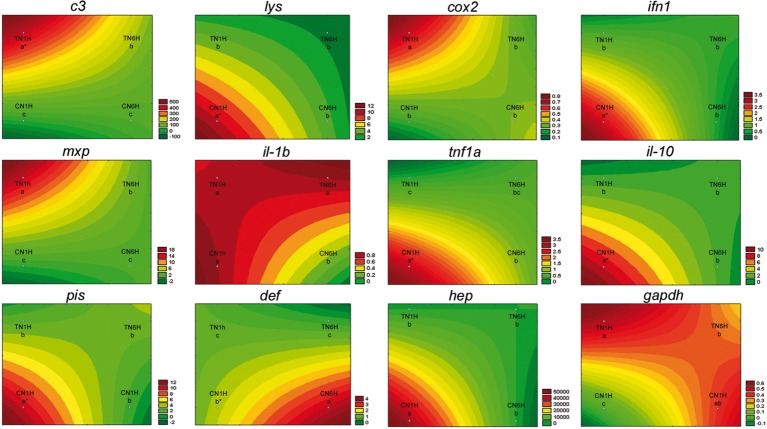
Visualization of gene expression results in gut-associated lymphoid tissue (GALT) after netting-handling stress at 1 and 6 h post-stress by means of response surface analysis. Quantitative PCR of specific mRNA markers of innate immune response processes (*c3 complement protein*, *lysozyme*, *cox2*, *mxp*, *piscidin*, *defensin*, and *hepcidin*), immune cytokines (*ifn*, *il-1b*, *tnf1a*, and *il-10*) and *gapdh,* normalized with elongation factor 1a and hypoxanthine-guanine phosphoribosyltransferase (*n* = 6 per experimental group). Asterisk shows differences between control without stress (resting fish) and control samples after 1 h of air exposure stress test without 1% Trp supplementation (REST analysis), whereas different letters denote statistically significant differences between experimental groups (two-way ANOVA). C, control feed; T, 1% Trp feed; N, netting stress; 1H/6H, 1/6 h post-stress.

Netting stress resulted in a high level of gene expression (asterisks) in *lys*, *ifn1*, *tnf1a*, *il-10*, *pis*, *def*, and *hep* genes in fish fed the C diet (Figure 3; REST analysis, *p* < 0.05). When considering the results of gene expression from fish fed the T diet, gene expression results for confinement/netting stress showed statistical differences at 1 hps (*p* < 0.05), with the highest and lowest expression values, at TN1H and TN6H, respectively, for *gapdh*, *c3 complement*, *cox2*, and *mxp*. On the other hand, the gene expression of *lys*, *ifn1*, *tnf1a*, *il-10*, *pis*, and *hep* was elevated when meagre was exposed to the confinement/netting stress, but only with the control diet at 1 hps (CN1H), whereas for the CN6H group (6 hps), *il-1b* showed a reduced expression and *def* a higher expression (Figure 4).

**Figure 5:**
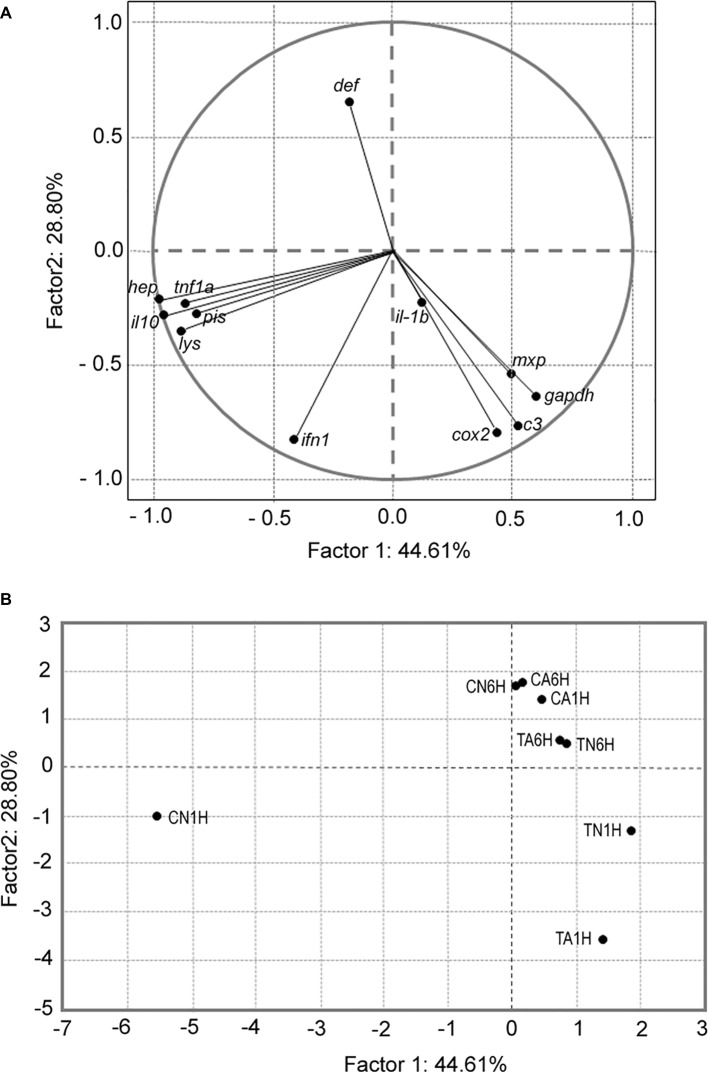
Principal components analysis of **(A)** gene expression according to the treatments set; *gadph*, *cox2*, *ifn1*, *il-1b*, *tnf1a*, *il-10*, *pis*, *def*, *hep, c3 complement*, *lys*, and *mxp*. **(B)** Correlation between treatment combination (tryptophan, time, and stress). Each dot represents the average of three biological replicate analyses of samples in the plot. Factors F1 and F2 used in this plot explain 73.40% of the total variance, which allows confident interpretation of the variation.

Results from the PCA (Figure 5A) showed two different groups of genes (*cox2*, *c3 complement*, *mxp*, and *gadph*) that were completely separated from the second one (*tnf1a*, *il-10*, *pis*, *hep*, and *lys*), whereas other genes like *ifn1*, *il-1b*, and *def* did not explain the relations between any dietary groups for either treatment. Results from PCA indicated that the first five components explained 99.5% of the cumulative variance; thus, the genes that better explained the association with the treatments were *gadph* (44.6%), *cox2* (28.7%), *ifn1* (15.4%), *il-1b* (8.6%), and *tnf1a* (2.0%). On the other hand, PCA between treatments also showed two groups: the first group included CA1H, TA6H, TN6H, CA6H, and CN6H, which was separated from the second group (TN1H and TA1H). Finally, fish from the treatment CN1H were totally separated from both groups (Figure 5B). According to this analysis, treatments that included 1% Trp (diet T) had a higher number of overexpressed genes from the second group (*tnf1a*, *il-10*, *pis*, *hep*, and *lys*) and they did not show any relation with those genes from the first group (*gadph*, *cox2*, *c3 complement*, and *mxp*). Additionally, fish fed the C diet and exposed to confinement/netting stress (CN1H) had a higher expression of genes from the second group (*tnf1a*, *il-10*, *pis*, *hep*, and *lys*).

**Figure 6:**
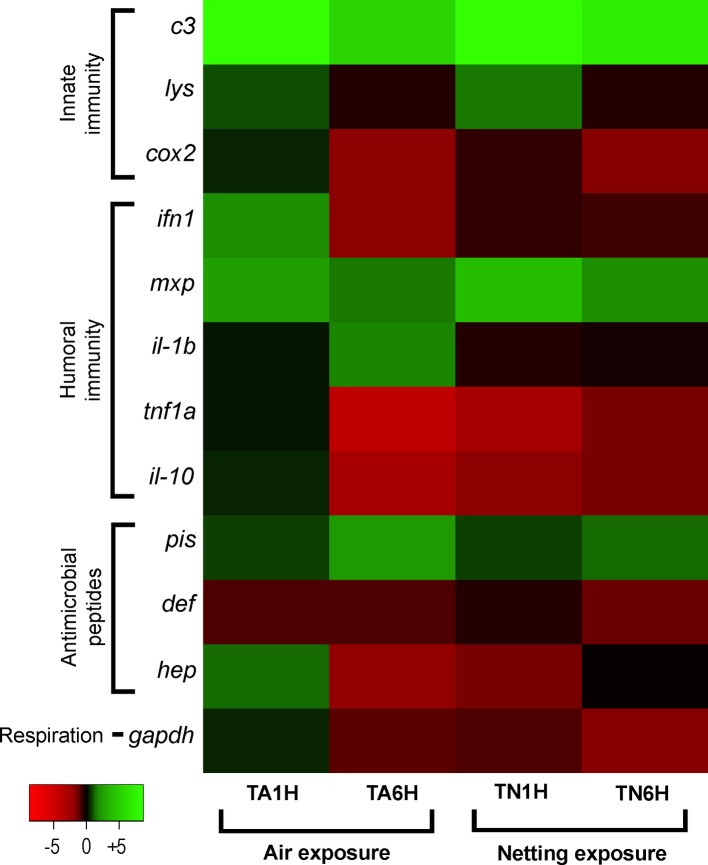
Heatmap image portraying expression patterns in gut-associated lymphoid tissue (GALT) after the air exposure and confinement/netting stress tests. Samples represented are from supplementation with 1% Trp and treated with air exposure (TA1H and TA6H) or confinement/netting exposure (TN1H and TN6H), and Control diet groups exposed to each stress (CA1H and CA6H for air exposure; CN1H and CN6H for netting). Changes in expression of innate immune response genes (*c3 complement protein*, *lysozyme*, *cox2*, *mxp*, *piscidin*, *defensin*, and *hepcidin*), immune cytokines (*ifn*, *il-1b*, *tnf1a*, and *il-10*) and *gapdh*, normalized with elongation factor 1a and hypoxanthine-guanine phosphoribosyltransferase, are represented in color: the green scale indicates downregulated genes and the red scale indicates induced genes.

Finally, the heatmap analysis clearly showed a higher gene expression pattern in meagre fed the Trp diet for air exposure at 1hps; by contrast, gene expression decreased under netting/confinement exposure after 1 and 6 hps (Figure 6).

## Discussion

Through commercial aquaculture activities (management protocols, vaccination protocols, crowding, and transportation, among others), fish are exposed to several stressors that threaten their welfare, survival, and the quality of the finished product(s). For these reasons, producers wish to alleviate stress during production to the extent that is possible (Galt et al., 2018). Barring elimination of stress, it would be beneficial to reduce the physiological response in fish to those stressors imposed upon them. As a consequence of external stressors, whether they are abiotic (hypoxia, temperature, osmotic shock, etc.) or biotic (predation, pathogens, handling, and transport by aquaculturists, among others), the stress manifests as a two-stage physiological response. The primary and secondary stress responses are well understood, and it is the release of endocrine factors during the primary response that leads to the secondary stress responses. The neuroendocrine pathways encompassed by the HPI and chromaffin sympathetic axes alter the blood chemistry in profound ways by the release of corticosteroids (Barton, 2002). This has immediate effects on all aspects of organ functioning *via* hormone receptors and secondary messenger systems, some of which involve specific gene transcription factors. In this study, we focused on the response of the anterior intestine to two different types of abiotic stressors: air exposure and netting/confinement. Since it has already been demonstrated that the stressors tested in the current study resulted in an increase in plasma cortisol levels in the same group of animals (Fernández-Alacid et al., 2019), herein we investigate the cascade of gene expression that follows the primary stress response in the intestine to understand whether the addition of L-tryptophan in the diet could alleviate the impact of handling stressors on the local gut immunity. Thus, the maintenance of a healthy gut, by alleviating some of the stress response, should have concomitant benefits for the overall health of the fish.

Results from the present study revealed that handling stressors affected local gut immunity as seen by changes in the gene expression patterns. In particular, meagre fed the C diet and stressed by air exposure for 3 min showed significant changes in the expression of several genes involved in innate immune responses (*c3*, *lys*, and *cox2*), humoral immune response processes (*il10*), and antimicrobial peptides (*def* and *hep*). In addition, stressing meagre fed the C diet by confinement and netting for 1 h resulted in significant changes in *lys*, *ifn1*, *tnf1a*, *pis*, *def*, and *hep*. These differences in the type and number of differentially expressed genes between both handling stressors may be due to their time of exposure (3 vs. 60 min with regard to air exposition and confinement/netting, respectively), as well as to the type of stressor. For instance, several studies have shown that the transcription factor hypoxia-inducible factor 1, which plays a major role in adaptive responses to hypoxia (Semeza, 1998), is also a mediator of immune functions (Hellwig-Bürgel et al., 2005; Palazón et al., 2014). This hypothesis may be supported by the fact that *cox2* was differentially expressed in fish stressed by air exposure, which agrees with some studies in higher vertebrates (Hellwig-Bürgel et al., 2005). However, the mechanisms by which different handling stressors effect the expression of local gut immune markers was not the objective of the present study; thus, this study does not provide conclusive results on this issue and the authors suggest that further research which is more specifically designed to address this issue is warranted.

Tryptophan is the only precursor of serotonin, whose function as a neurotransmitter plays a key role in reducing stress and aggression, acting through the HPI axis to control osmoregulatory, hematological, immunological, and behavioral responses (Wen et al., 2014). Several studies have shown that supplementation of feed with small amounts of Trp reduces stress in various cultivated species and improves their immune response (Andersen et al., 2016). The results obtained in the present study showed that feeding meagre with a diet supplemented with 1% Trp for 7 days resulted in an upregulation of gene expression at 1 hps in the air exposure stress group for most of the gut immune genes evaluated, such as *c3*, *lys*, and *cox2* (innate immune system response), *ifn1*, *mxp*, and *il-10* (humoral immune system), *pis* (antimicrobial peptides), and *gapdh* (mitochondrial respiratory). On the other hand, the confinement/netting stress test exhibited its primary effect in fish fed the T diet only in expression of *c3* and *mxp* genes. As previously suggested, these results indicate that different stressors may have different effects on local gut immunity (Hellwig-Bürgel et al., 2005; Palazón et al., 2014), as well as demonstrating the positive effects of Trp dietary supplementation. These results from supplementation of Trp are due in part to it being provided as a free-tryptophan where, after its absorption, it is converted to serotonin by the enzyme tryptophan hydroxylase. Serotonin is involved in the modulation of the endocrine system and serum cortisol levels (Lim et al., 2013; O’Mahony et al., 2015). It has been estimated that in higher vertebrates, *ca.* 95% of serotonin is found in the gastrointestinal tract (Kim and Camilleri, 2000). Although serotonin was not measured in the brain nor in the gut of meagre in the current study, higher levels of cortisol in fish exposed to both handling stressors (Fernández-Alacid et al., 2019) seemed to validate the former postulate and confirmed our results in meagre fed diets supplemented with Trp. In our study, fish fed the 1% Trp supplementation and treated with the air exposure stress test showed an upregulation of genes involved in innate (*c3*, *lys*, and *cox2*) and humoral (*ifn1*, *mxp*, and *il-10)* responses. However, the gene expression response was not so clear for fish exposed to the confinement/netting test, which may be due to different types of physiological responses to brief hypoxia versus chasing with a net (Hellwig-Bürgel et al., 2005; Palazón et al., 2014). In this context, the importance of the abovementioned genes (*cox2*, *ifn1*, *il-1b*, *tnf1a*, and *gadph*) in the stress response in meagre fed the 1% Trp diet was corroborated by the PCA results, as those genes explained more than 73% of the total variance observed. Considering the abovementioned results, air exposure and netting/confinement stressors may affect the biological homeostasis of serum Trp due to its involvement in serotonin synthesis (Lim et al., 2013), and consequently, these stressors can induce changes in metabolic, hematological, hydromineral, and structural responses (Barton and Iwama, 1991). Under present experimental condition, fish fed the C diet had elevated levels of circulating corticosteroids (Fernández-Alacid et al., 2019), which may reduce immuno-competence by influencing lymphocyte numbers and antibody-production capacity, which function in adaptive immune memory against pathogens, and changes in the external environment (Hoseini et al., 2012). Present results revealed that an adequate dietary supplementation of Trp generated an upregulation of genes from the innate and humoral immune systems, which would stimulate the gut-associated lymphoid tissue (GALT) and activate the immune system, providing protection to the host against potential pathogen invasions, as has been widely documented in other fish species (Dinan and Cryan, 2012; Lazado and Caipang, 2014; O’Mahony et al., 2015). In agreement with present results, a study conducted in European sea bass (*Dicentrarchus labrax*) fed diets supplemented with 0.5% of Trp and challenged with an intraperitoneal injection of *Photobacterium damselae* subsp. *Piscicida* demonstrated that immune-related genes were upregulated in the gut (Azeredo et al., 2017b). The abovementioned effect could not be corroborated in *A. regius* because no bacterial challenge was conducted in this study; however, our data on local gut immunity supported the hypothesis of a positive effect of this amino acid on the overall condition and immunity of fish.

In the present study, meagre fed the commercial diet supplemented with 1% Trp showed an upregulation of *c3* in both stress tests. Moreover, innate immune functions performed by *lys* expression only increased in fish exposed to the air stress after 1 hps (TA1H), while for confinement/netting stress, *lys* expression was not modified by the addition of Trp to the diet. The expression of c3 complement protein functions toward removal of foreign bodies such as viral particles and bacteria by opsonization and the induction of bacterial lysis, thereby enabling targeted foreign organisms to be destroyed by phagocytes (Rooijakkers and van Strijp, 2007). C3 complement also participates in inflammatory reactions (Raftos et al., 2003) and functions as a modifier of acquired immunity (Sunyer et al., 1998; Claire et al., 2002; Lange and Magnadóttir, 2003; Ricklin et al., 2010). While lysozyme plays an important role functioning as a lytic enzyme by hydrolyzing the peptidoglycan component of bacterial cell walls (Davis and Weiser, 2011), thereby limiting bacterial infections and further activating the complement system and phagocytes (Paulsen et al., 2003); thus, these two innate effectors work together to clear infections. Since lysozyme is less specific in its action as it is not influenced by acquired immunity in the same way as the complement system (Claire et al., 2002), it would make sense that this molecular effector is more tightly controlled for preventing unwanted damage to the commensal microbiota of the gut (Gao et al., 2018).

In the air exposure stress test, fish fed with 1% Trp supplementation demonstrated an upregulation of gene expression at 1 hps (TA1H) of *c3*, *lys*, and *cox2*, although for the confinement/netting stress test, there was downregulation of expression for *cox2*. Prostaglandin endoperoxide H synthase, or *cox2*, functions in oxygenating a wide range of fatty acid and fatty esters (Vecchio et al., 2010), leading to the induction of inflammation *via* prostaglandin production (Dyall, 2017). The contrasting response in *cox2* expression with the two different stress exposure treatments may also be related to alternate pathways of gene signaling *via* transcription factors such as hypoxia-inducible factor 1alpha (HIF1a) and HIF1B (Pelster and Egg, 2018).

Regarding humoral immune response processes, our study showed that the supplementation of a commercial diet with 1% Trp increased the expression of *ifn1*, *mxp*, *tnf1a*, and *il-10* at 1 hps when fish were subjected to an air exposure stress test, while their expression decreased at 6 hps. By contrast, the confinement/netting stress test only resulted in an upregulation of *mxp* at 1hps. These results agree with a study in barramundi (*Lates calcarifer*) fed a diet supplemented with 1% Trp where there was high lysozyme activity and an upregulation of *mxp* expression after a challenge with nervous necrosis virus (NNV) (Chi et al., 2018). These results may be explained by the participation of interferon regulatory factors (IRF) in the regulation of the abovementioned proteins. IRFs are a group of transcription factors whose synthesis requires Trp (Mamane et al., 1999), as they contain a characteristic repeat of five tryptophan residues in a DNA-binding domain. Through this DNA-binding domain, IRF family members bind to a similar DNA motif, termed IFN stimulated response element (ISRE). It follows that when the free serum Trp is increased by a dietary supplement, there can be expected an increase in synthesis of IRF with down-stream effects on expression of immune genes such as *ifn1* and *mxp*. Among the molecular effectors examined herein, type I interferon (*ifn1*) can stimulate cellular effectors like natural killer cells and macrophages (Kvamme et al., 2013). In addition, *ifn1* positively regulates the *mxp* antiviral protein, whose coding sequence may be regulated by the presence of ISREs (Das et al., 2008). The increase in *ifn1* expression in meagre subjected to air exposure stress test at 1 hps (TA1H) could be explained by the abovementioned results. However, the confinement/netting stress test did not evidence this same expression pattern, as in the case of *cox2*, and it may be related to differences in transcriptional signaling.

The expression of *il-1b* increased at 6 hps for fish fed 1% Trp under air exposure stress (TA6H); however, when considering the confinement/netting stress test this gene had the lowest expression at 1 and 6 hps (TN1H and TN6H respectively) together with *ifn1*, *il-1b*, *tnf1a*, and *il-10*. Given these results, it seems to be that confinement/netting test is not so aggressive a stressor as the air exposure test. Among the cytokines studied herein, interleukin-1B (*il-1b*) influences the function of the HPI axis by stimulating the secretion of cortisol (Engelsma et al., 2002; Holland and Lambris, 2002). Additionally, *tnfα* is a significant pro-inflammatory cytokine produced by a variety of lymphocytes that promotes apoptosis and macrophage respiratory burst activity and can promote antiviral response *via il-1b* stimulation (Qin et al., 2001). It could be possible that in our study these pro-inflammatory cytokines responded in different ways to each stressor due to feedback or influence of other cytokines not presented in this study. In general, gene expression of the pro-inflammatory cytokines examined seemed to have a tendency to be influenced by the addition of 1% Trp in the diet; however, more research is necessary to elucidate this aspect.

With regard to cytokines, Trp can function as a first step in signaling T cell proliferation and this may be functioning in part due to the ISREs already mentioned (Jenkins et al., 2016). Once activated, different classes of T cells produce additional cytokines that have a variety of effects on a broad range of immune functions, some of which are more anti-inflammatory than pro-inflammatory. Interleukin 10 functions to modulate inflammatory responses. In this study under the influence of air exposure, *il-10* expression was upregulated at 1 hps (TA1H) in fish fed the 1% Trp supplementation, whereas for the same dietary treatment in the confinement/netting stress groups TN1H and TN6H, *il-10* showed a downregulation in expression, which could indicate a potential positive effect of Trp on gut condition. Ordinarily, *il-10* is associated with an anti-inflammatory response. Under a dietary supplement of 1% Trp and confinement/netting stress, depression of *il-10* expression would enable a more robust pro-inflammatory response, potentially benefitting the host. Although under such conditions where *il-10* is downregulated, there may be over-expression of inflammatory cytokines and activation of natural killer cells that could be a source of unwanted tissue damage, which has been detected in *D. labrax* fed 0.52% Trp supplementation (Azeredo et al., 2017c).

In the gut, antimicrobial peptides are expressed somewhat continuously to maintain a balance among commensal versus potentially pathogenic bacteria (Ostaff et al., 2013). This is achieved through what are likely tight controls over their expression to prevent unwanted damage to the host microbiota. In this study, *pis* was upregulated at 6 hps in meagre fed the Trp diet and subjected to air exposure stress (TA6H), and this tendency was already seen at 1 hps (TA1H), even though it was not statistically significant. By contrast, *pis* expression was not affected by the same dietary treatment when fish were exposed to the confinement/netting stress test. Furthermore, *hep* and *def* expression in fish fed the 1% Trp diet and subjected to the air exposure test had a tendency toward reduced expression values at both sampled times (1 and 6 hps), even though no statistical differences were found due to high inter-individual variability. For fish exposed to the confinement/netting stress and fed the 1% Trp diet, we found *def* to be downregulated at both sampling times (1 and 6 hps). The alterations of expression of AMPs may be a cause of imbalance to the host microbiota (Ostaff et al., 2013), although since the expression of AMPs was different depending on the gene in question and the handling stressor applied no specific conclusions can be drawn about a general effect. Different stressor signals are involved since the response was markedly different for *pis* as compared to *hep* and *def* at 1 hps depending on the type of stressor (air exposure versus confinement/netting). Antimicrobial peptides, such as defensin, piscidin, and hepcidin proteins, are important effector molecules of the innate immune system (Campoverde et al., 2017) that are thought to function by lytic mechanisms (Campagna et al., 2007; Noga et al., 2009; Smith et al., 2010). Depending on the class of AMP considered, they can be effective against viral, fungal, or bacterial pathogens (Lauth et al., 2005; Falco et al., 2008; Casadei et al., 2009; Masso-Silva and Diamond, 2014; Campoverde et al., 2017). Stimulation using pathogen-associated molecular patterns (PAMPs) has been shown to specifically increase their expression under experimental trials (Casadei et al., 2009; Campoverde et al., 2017). Piscidin also has the capacity to modulate gene expression of pro-inflammatory and immune-associated genes (Lin et al., 2016; Yang et al., 2016). The capacity for *pis* to modulate expression of pro-inflammatory immune genes may explain the different patterns of expression seen between *pis* and the other two AMPs analyzed in the current study, or the downregulation of AMPs observed under the air exposure stress conditions applied in this study may be related to the upregulation of *il-10*. Keeping the AMP expression under multiple regulatory controls likely aids in preventing dysbiosis of the gut due to lysis of beneficial members of the host microbiota (Ostaff et al., 2013).

Finally, it is worth mentioning that under confinement/netting stress, the addition of Trp augmented the expression of *gapdh*. This increase in *gapdh* expression could provide a benefit for energy production, and therefore, improvements in all other metabolic events related to the animal’s energy balance, which has been demonstrated *in vitro* using intestine cell cultures from European sea bass *D. labrax* supplemented with Trp (Azeredo et al., 2017c). This increment in *gapdh* gene expression was observed in meagre as a response of both stressors after 1 hps in fish fed the Trp diet in comparison to the control group. This gene was initially considered as a housekeeping gene in our analyses, but when checking its level of expression, the authors found that it was differentially regulated depending on the stressor considered, which may be attributed to this enzyme’s function in cellular respiration. The transfer of electrons during respiration requires NAD^+^ as an electron carrier; Trp is a metabolic precursor and requirement for NAD^+^ synthesis. Therefore, Trp availability may be a rate-limiting point in the transfer of electrons and the functioning of respiration. With more availability of Trp, there is the possibility of an increase in the synthesis of NAD^+^ (Richard et al., 2009), relieving this rate-limiting step in respiration. The increase in respiration would be made evident by an increase in the production of the key enzymes in this pathway, such as *gapdh*. Considering these results, special attention should be paid when using *gadph* as housekeeping genes in studies were fish may be exposed to potential stressing conditions.

## Conclusions

The two handling stressors tested in this study, which are quite common under standard aquaculture practices, resulted in some distinctly different gene expression responses depending on the type of stress, the dietary supplementation of Trp, and the recovery time (time post-stress). Some of the differences observed in gene expression may be related to positive or negative feedback mechanisms, such as *Il-10* signaling of AMPs. Air exposure may have an important effect distinguishing the two responses. The differences between the two stress responses may be related to specific transcriptional regulatory pathways of local gut immune genes, such as HIFs, that will require further study to confirm. The inclusion of 1% Trp in diets for *A. regius* had clear pronounced effects on physiological functions, as would be expected since this particular supplement implicates the HPI and chromaffin sympathetic axes, which influence vital physiological functions. The stresses imposed in this study were meant to mimic those generated during fish aquaculture practices that are known to increase serum cortisol levels. The overall effect of the Trp supplemented diet on the mucosal immunity of the gut seemed to allow a more balanced response, alleviating some of the more damaging aspects of the stress response, such as elevated AMP expression that could change the microbiota and have long-term negative effects such as outbreaks of opportunistic bacterial pathogens, although this needs to be studied further using specific challenge experiments. Moreover, there was seen a significant increase in the nonspecific innate immune molecules *ifn*, *mxp*, and *c3* that should help to prime the immune system in a nonspecific manner against infections. Lastly, the energy balance would seem likely to benefit from an increase in the expression in *gapdh* that is related to the requirement for tryptophan as a substrate for NAD^+^ synthesis. Further work is needed to understand the additional functions of Trp on gut health for the benefit of cultured fish species and confirm the potential mechanisms observed herein.

## Author Contributions

EG and MH were the scientific leaders and project supervisors, and designed the experiments presented in this paper. KA designed the gene expression assays, directed the molecular analyses, and revised the English language. IG performed the *in vivo* experiment, and GA-A conducted the molecular analyses. Finally, all authors read and contributed in the preparation and revision of the final manuscript.

### Conflict of Interest Statement

The authors declare that the research was conducted in the absence of any commercial or financial relationships that could be construed as a potential conflict of interest.
